# Serum proteomes and their prognostic values in sepsis patients admitted to a medical intensive care unit: a single-center study using SWATH-MS proteomics

**DOI:** 10.1186/s13613-025-01543-y

**Published:** 2025-08-27

**Authors:** Su Yeon Lee, Jee Hwan Ahn, Sang-Bum Hong, Dong-Gon Hyun, Chae-Man Lim, Kyunggon Kim, Jin Won Huh

**Affiliations:** 1 Department of Pulmonary and Critical Care Medicine, Asan Medical Center, University of Ulsan College of Medicine, 88, Olympic-ro 43-gil, Songpa-gu, Seoul, 05505 South Korea; 2 Department of Convergence Medicine, Asan Medical Center, Asan Institute for Life Sciences, University of Ulsan College of Medicine, 88, Olympic-ro 43-gil, Songpa-gu, Seoul, 05505 South Korea

**Keywords:** Sepsis, Proteomics, Biomarkers, Prognosis, Intensive care units

## Abstract

**Background:**

Recent developments in proteomics suggest opportunities to understand the pathophysiological heterogeneity of sepsis and provide precision medicine tailored to individual patients. This study aims to evaluate the serum proteomic profiles of patients with sepsis using Sequential Window Acquisition of All Theoretical Mass Spectra proteomics and identify novel biomarkers for assessing sepsis severity and predicting patient outcomes in the ICU.

**Methods:**

This retrospective cohort study included 217 adult patients diagnosed with bacterial sepsis who were admitted to a medical ICU at a single tertiary hospital between January 2011 and January 2020, along with 292 healthy controls. Proteomic analysis was performed to compare patients with sepsis to the 292 healthy controls and analyze differences among sepsis subgroups. The subgroups were classified according to their outcome: early death (within 3 days), late death (after 3 days), and recovery (survived and discharged).

**Results:**

Five key proteins—β-actin (ACTB), fibronectin (FINC), metalloproteinase inhibitor 1 (TIMP1), platelet factor 4 (PF4), and C-X-C motif chemokine 7 (CXCL7)—were identified as significant discriminators of sepsis subgroups with AUCs ranging from 0.786 to 0.833. A six-variable model including 5 proteins and the SOFA score showed the highest AUC value of 0.903 for predicting in-hospital mortality. Multivariable Cox proportional hazards analysis revealed that increased ACTB (hazard ratio [HR] 1.21 [1.07–1.36], *p* = 0.002), decreased FINC (HR 0.88 [0.79–0.98], *p* = 0.024), higher SOFA scores (HR 1.08 [1.03–1.13], *p* = 0.002) and gram-negative sepsis (HR 1.42 [1.02–1.97], *p* = 0.038) were significantly associated with increased in-hospital mortality.

**Conclusions:**

A novel set of biomarkers was identified in this study, including ACTB, FINC, CXCL7, TIMP1, and PF4, for assessing sepsis prognosis. The six-variable model incorporating SOFA scores demonstrated a high prognostic value for in-hospital mortality, potentially enabling more accurate risk stratification for patients with sepsis in the ICU.

**Supplementary Information:**

The online version contains supplementary material available at 10.1186/s13613-025-01543-y.

## Background

Sepsis is a life-threatening condition characterized by organ dysfunction resulting from a dysregulated host response to infection [1]. Approximately one-third of critical illness patients admitted to the intensive care unit (ICU) are diagnosed with sepsis [2]. Although advances in critical care management have reduced mortality rates [3], patients with septic shock still experience poor clinical outcomes in the ICU [2, 4]. Early prediction of mortality risk in patients with sepsis is crucial for optimizing patient management and improving outcomes [5]. Several clinical scoring systems, such as the Sequential Organ Failure Assessment (SOFA), the Acute Physiology and Chronic Health Evaluation, and the Simplified Acute Physiology Score, have been developed to assess patient severity and predict ICU mortality [6–8]. For instance, the SOFA score calculated in the first few days of ICU admission based on organ dysfunction severity is a strong predictor of prognosis [6, 9]. However, the complexity of certain scoring systems, which depend on multiple clinical and laboratory variables, limits their real-time application and introduces variability due to subjective components [10]. In contrast, biomarkers are objective and measurable indicators, prompting studies to investigate potential biomarkers for patients with sepsis [11]. However, biomarkers for predicting the outcome of patients with sepsis remain limited.

Proteomics is a valuable tool for identifying protein expression and changes associated with disease, providing insight into potential biomarkers for diagnosing and evaluating patients with sepsis [12]. Sepsis is a complex, heterogeneous condition driven by variations in the immune response of the host. Proteomics can provide insights into this pathophysiological heterogeneity of sepsis and offer personalized or precision medicine for individual patients. The Sequential Window Acquisition of All Theoretical Mass Spectra (SWATH-MS), a promising mass spectrometry technique for biomarker discovery, is highly effective for the analysis of proteins in human body fluids [13, 14]. Therefore, this study aims to evaluate the proteomic profiles of patients with sepsis using SWATH-MS-based proteomics and identify novel biomarkers for diagnosing sepsis and predicting patient outcomes across sepsis subgroups.

## Materials and methods

### Study design and patients

This retrospective cohort study was conducted using data from a sepsis registry at a single tertiary hospital in Korea. The study included adult patients with sepsis (age > 18 years) who were admitted to the medical ICU between January 2011 and January 2020. Patients were included in the registry if they met the following criteria: (1) admission to the medical ICU with a sepsis diagnosis according to the Sepsis-2 or Sepsis-3 definitions [1, 15], and (2) consent to enroll in the sepsis registry and provide blood samples. Patients who declined participation in this registry were excluded. Only patients with confirmed bacterial infections were included in the analysis. Time zero was defined as the date of registry enrollment and blood sample collection. A control group of 292 healthy individuals without active diseases was selected from the registry of the Health Screening and Promotion Center at Asan Medical Center. The study included two distinct analyses. First, a comparison of the proteomic profiles of patients with sepsis with those of healthy controls; Second, an investigation into the differences in the proteomic profiles among sepsis subgroups based on their clinical outcomes. Sepsis subgroups were predefined based on their outcomes: the early death (patients who died within 3 days of enrollment), late death (patients who died 3 days after enrollment), and recovery groups (patients who survived and were discharged). This definition was adapted from a previous study that defined early death as occurring within the first 3 days of ICU admission, primarily due to refractory organ failure associated with the primary infection [16]. Patients discharged to hospice were recorded as deceased on the day of discharge.

### Data collection, processing and statistical analyses

The following patient data were collected from the electronic medical records: age, sex, height, weight, body mass index, underlying comorbidities, hospital and ICU admission and discharge dates, sample collection date, date of death, pathogen type, infection site, highest SOFA score, and lactate levels on the day of study enrollment, and use of mechanical ventilation, renal replacement therapy, extracorporeal membrane oxygenation, or adjuvant steroids during ICU admission. Serum samples from patients with sepsis and healthy controls were analyzed using SWATH-MS. Detailed protocols for proteomic sample collection, preparation and SWATH-MS analysis are described in Appendix 1. Statistical methods for processing proteomic data and analyzing clinical variables are provided in Appendix 2.

## Results

**Fig. 1:**
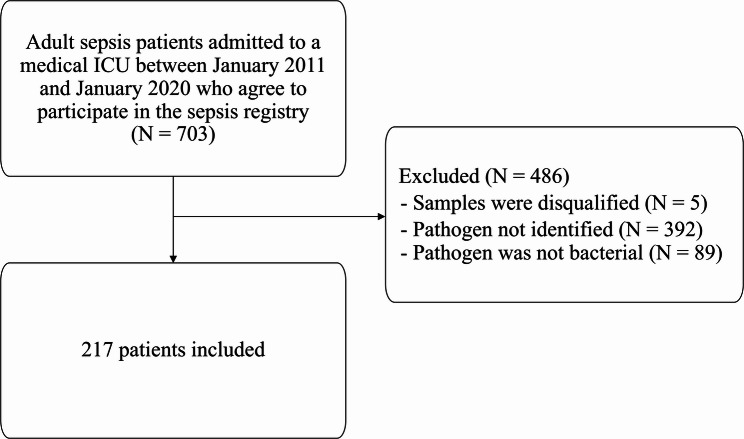
Patient inflow diagram. Abbreviations: ICU, intensive care unit

This study included 217 patients diagnosed with bacterial sepsis (Fig. 1). The median age of the patients was 64.0 years (IQR, 54–72), with 70% being male (Table 1). The most common site of infection was the pulmonary system (53.5%). The mean SOFA score and lactate level were 13.4 ± 4.7 and 4.7 ± 4.4 mmol/L, respectively. Of the 217 patients with sepsis, 17.5% (38 patients), 54.4% (118 patients), and 28.1% (61 patients) were in the early death, late death, and recovery group, respectively. The early death group had a median age of 55 years (IQR, 44–70) and exhibited the highest SOFA scores (17.0 ± 3.7) and lactate levels (8.4 ± 4.6 mmol/L) on enrollment day (*p* < 0.001 for both). Serum procalcitonin levels did not differ significantly between the subgroups (*p* = 0.384). Among the 292 healthy controls, the median age was 50.5 years (IQR, 31–61), with 69.1% being male.

**Table 1:** Baseline characteristics and ICU treatments of sepsis groups

	Early death (*N* = 38)	Late death (*N* = 118)	Recovery (*N* = 61)	Total (*N* = 217)	*P*-value
Age, median (IQR)	55 (44–70)	64 (57–72)	66 (58–74)	64 (54–72)	0.035
Male, n (%)	26 (68.4)	79 (66.9)	47 (77.0)	152 (70.0)	0.365
BMI, mean ± SD	23.2 ± 3.6	22.8 ± 3.5	22.6 ± 3.9	22.8 ± 3.6	0.712
Comorbidity, n (%)					
Cardiovascular disease	3 (7.9)	18 (15.3)	9 (14.8)	30 (13.8)	0.505
Chronic lung disease	4 (10.5)	15 (12.7)	8 (13.1)	27 (12.4)	0.923
Chronic neurologic disease	0 (0.0)	10 (8.5)	11 (18.0)	21 (9.7)	0.010
Chronic liver disease	15 (39.5)	37 (31.4)	7 (11.5)	59 (27.2)	0.003
Diabetes	11 (28.9)	40 (33.9)	18 (29.5)	69 (31.8)	0.767
Connective tissue disease	3 (7.9)	3 (2.5)	2 (3.3)	8 (3.7)	0.307
Hematological malignancies	10 (26.3)	21 (17.8)	2 (3.3)	33 (15.2)	0.004
Solid malignant tumors	10 (26.3)	48 (40.7)	22 (36.1)	80 (36.9)	0.277
Source of infection, n (%)					
Pulmonary	21 (55.3)	72 (61.0)	23 (37.7)	116 (53.5)	0.012
Abdominal	10 (26.3)	24 (20.3)	18 (29.5)	52 (24.0)	0.369
Urinary	2 (5.3)	1 (0.8)	8 (13.1)	11 (5.1)	0.002
Skin soft tissue	3 (7.9)	8 (6.8)	6 (9.8)	17 (7.8)	0.771
Catheter	0 (0.0)	3 (2.5)	1 (1.6)	4 (1.8)	0.593
Systemic	2 (5.3)	10 (8.5)	3 (4.9)	15 (6.9)	0.611
Central nervous system	0 (0.0)	2 (1.7)	1 (1.6)	3 (1.4)	0.724
Endocarditis	2 (5.3)	2 (1.7)	2 (3.3)	6 (2.8)	0.486
SOFA score, mean ± SD	17.0 ± 3.7	14.3 ± 3.7	9.4 ± 4.1	13.4 ± 4.7	< 0.001
WBC, ×10^3^/uL, mean ± SD					
Platelet, ×10^3^/uL, mean ± SD	57.2 ± 63.7	90.1 ± 89.3	135.9 ± 93.0	97.2 ± 90.3	< 0.001
Lactate, mg/dL, mean ± SD	8.4 ± 4.6	4.8 ± 4.5	2.3 ± 2.0	4.7 ± 4.4	< 0.001
Procalcitonin, ng/mL, mean ± SD	16.2 ± 23.6	30.3 ± 51.3	37.8 ± 54.9	31.9 ± 50.6	0.384
Gram negative pathogen, n (%)	27 (71.1)	60 (50.8)	30 (49.2)	117 (53.9)	0.064
Bacteremia, n (%)	23 (60.5)	67 (56.8)	41 (67.2)	131 (60.4)	0.4
Septic shock, n (%)	30 (78.9)	96 (81.4)	41 (67.2)	167 (77.0)	0.098
ICU treatment, n (%)					
Mechanical ventilation	33 (86.8)	110 (93.2)	26 (42.6)	169 (77.9)	< 0.001
Renal replacement therapy	24 (63.2)	69 (58.5)	11 (18.0)	104 (47.9)	< 0.001
Adjuvant steroids	26 (68.4)	87 (73.7)	18 (29.5)	131 (60.4)	< 0.001
Sampling day from ICU admission, median (IQR)	1 (0–2)	1 (0–2)	1 (1–1)	1 (0–2)	0.328

Abbreviations: IQR, interquartile range; SOFA, sequential organ failure assessment; WBC, white blood cell; ICU, intensive care medicine

**Fig. 2:**
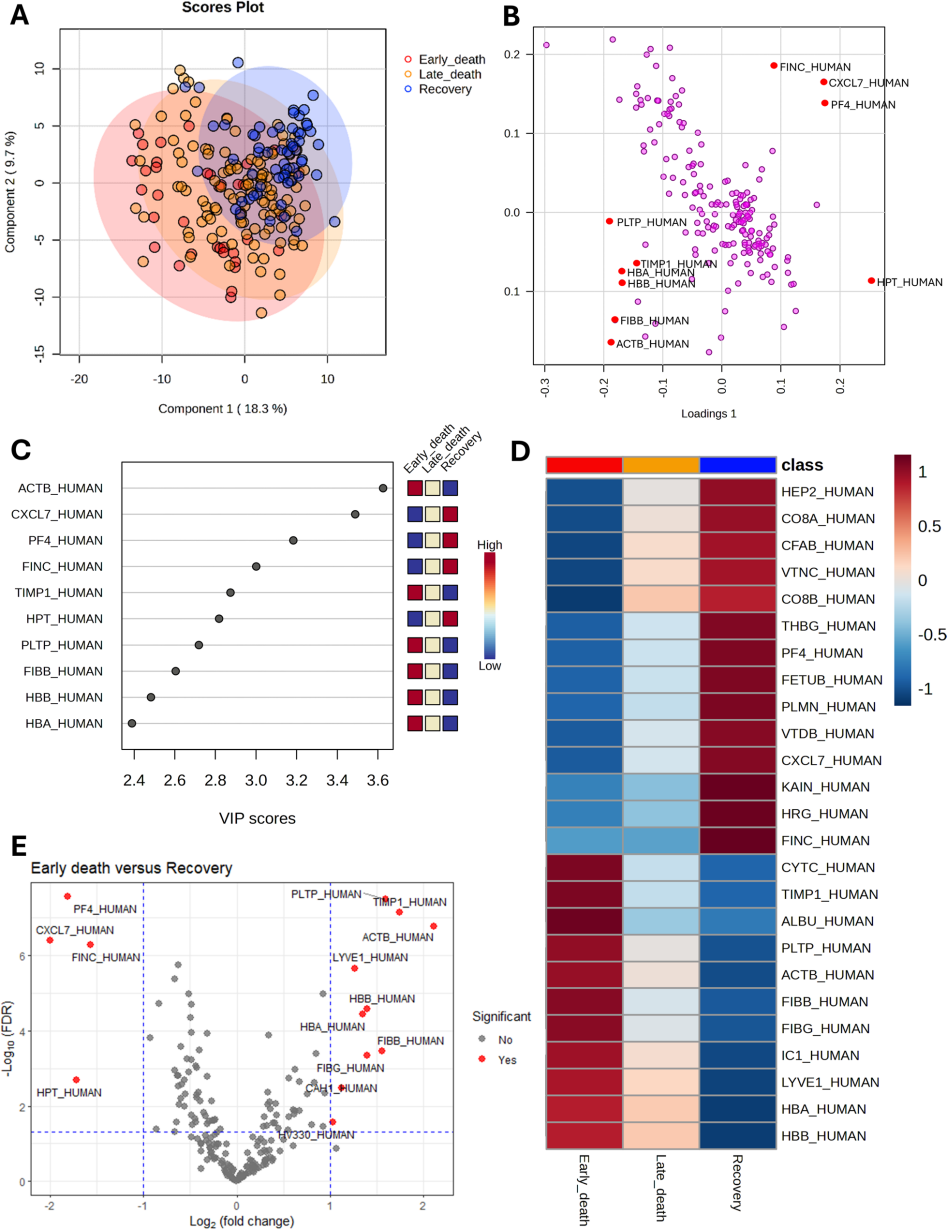
Proteomic profiles of patients with sepsis across outcome-based subgroups. (**A**) PLS-DA based on protein abundance in samples from patients with sepsis. A gradient was observed along Components 1 and 2, progressing from the early death group to the late death group and then to the recovery group. A distinct separation was observed between the early death and recovery groups, while the late death group exhibited characteristics that were a blend of the early death and recovery groups. (**B**) PLS-DA loading plot for components 1 and 2. In the loading plot, β-actin (ACTB) showed negative loading for both components, while fibronectin (FINC), C-X-C motif chemokine 7 (CXCL7), and platelet factor 4 (PF4) exhibited high positive loadings for both components. (**C**) VIP scores for the top 10 proteins. The top 5 proteins with the highest VIP scores, which accounted for the differences between sepsis groups, were ACTB, CXCL7, PF4, FINC, and Metalloproteinase Inhibitor 1 (TIMP1). (**D**) Hierarchical heatmap depicting the top 25 discriminating proteins. The heatmap comparing sepsis subgroups revealed distinct expressions of the five proteins—ACTB, CXCL7, PF4, FINC, and TIMP1—across the subgroups, with their mean values correlating with sepsis outcomes. (**E**) Volcano plot illustrating differential protein abundance between patients with sepsis with early death and those who recovered. The volcano plot indicated that ACTB and TIMP1 were expressed at higher levels in the early death group, while PF4, CXCL7, and FINC expression levels were higher in the recovery group. Abbreviations: PLS-DA, partial least squares discriminant analysis; VIP, variable importance in projection

The results of proteomic profiles of sepsis groups compared to healthy controls were described in Figure S1. Acute phase reactants, including c-reactive protein (CRP), serum amyloid A-1 (SAA1), and serum amyloid A-2 (SAA2), significantly discriminated sepsis patients from healthy controls. When comparing sepsis subgroups based on their outcomes, five key protein markers—β-actin (ACTB), C-X-C motif chemokine 7 (CXCL7), platelet factor 4 (PF4), fibronectin (FINC), and Metalloproteinase Inhibitor 1 (TIMP1)—were identified as significant discriminators (Fig. 2). Table S1 lists all the proteins that were significantly different according to ANOVA, along with the post hoc analysis results between sepsis subgroups. While CRP, SAA1, and SAA2 did not show significant differences between sepsis subgroups, the five proteins differed across the sepsis subgroups. Figure S2 presents box plots showing the protein abundance of CRP and the five proteins across the healthy controls, early death, late death, and recovery groups, demonstrating consistent trends in their levels.

**Fig. 3:**
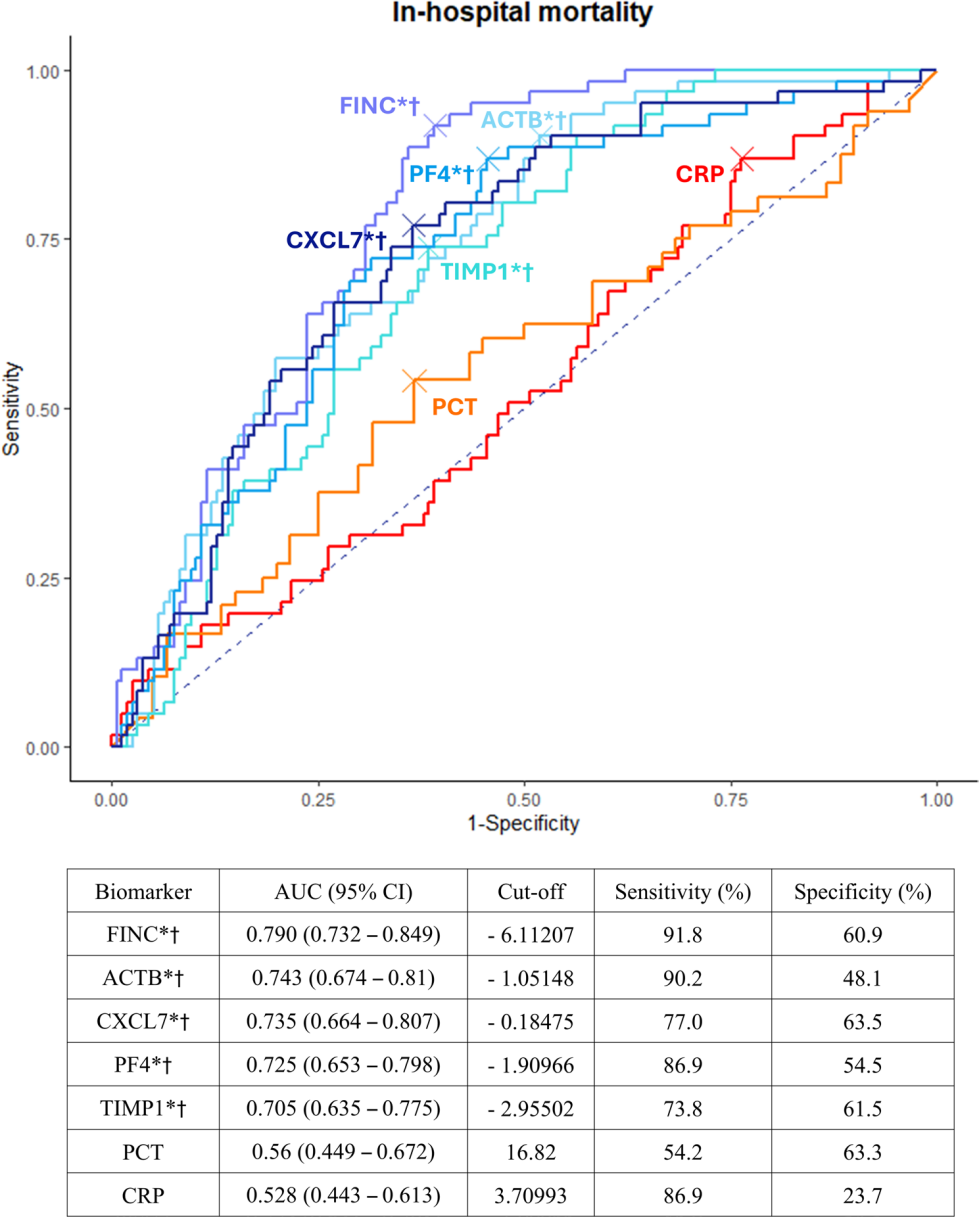
ROC curves for prediction of in-hospital mortality. * indicates a significant difference (*p* < 0.05) compared to the CRP ROC curve according to DeLong’s test, and † indicates a significant difference (*p* < 0.05) compared to the PCT ROC curve. Abbreviations: ROC, receiver operating characteristic; AUC, area under the curve; PCT, procalcitonin; CRP, C-reactive protein; ACTB, β-actin; FINC, fibronectin; CXCL7, C-X-C motif chemokine 7; PF4, platelet factor 4; TIMP1, Metalloproteinase Inhibitor 1

**Fig. 4:**
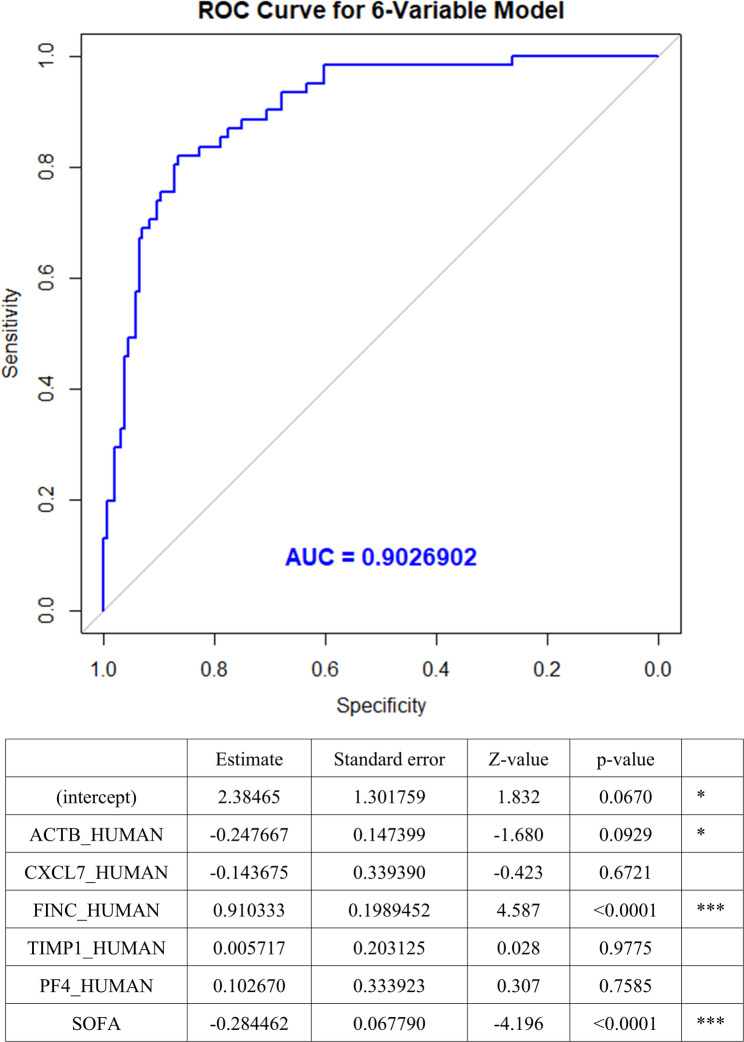
ROC curves for the optimal six-variable model in predicting in-hospital mortality with the highest AUC value (0.903). In this model, the sensitivity and specificity at the best threshold were 82.0% and 86.5%, respectively. Abbreviations: ROC, receiver operating characteristic; AUC, area under the curve; ACTB, β-actin; FINC, fibronectin; CXCL7, C-X-C motif chemokine 7; PF4, platelet factor 4; TIMP1, Metalloproteinase Inhibitor 1

We analyzed the area under the curve (AUC) values for each protein to distinguish between healthy controls and patients with sepsis, as well as among sepsis subgroups (Figure S3). The five proteins—ACTB, CXCL7, PF4, FINC, and TIMP1— demonstrated good discriminatory ability between patients with sepsis subgroups and healthy controls, especially between early death and recovery, with AUC values ranging from 0.786 to 0.833 (all, *p* < 0.001). Figure 3 shows the AUC curves and cut-off values for predicting in-hospital mortality alongside the predictive values for CRP, the five proteins, and serum procalcitonin. The five proteins demonstrated higher predictive values for in-hospital mortality than serum procalcitonin and CRP, with AUC values ranging from 0.705 to 0.790, respectively (all, *p*-value < 0.05). To enhance the prediction of in-hospital mortality, models were developed by combining five key proteins with the SOFA score. The five-protein model incorporating ACTB, FINC, CXCL7, TIMP1, and PF4 achieved an AUC of 0.867, while the SOFA and lactate model yielded an AUC of 0.839 (Figure S4). The six-variable model incorporating ACTB, FINC, CXCL7, TIMP1, PF4, and SOFA was identified as the most effective, achieving the highest AUC value of 0.903 (Fig. 4). At the optimal threshold, the model demonstrated a sensitivity of 82.0% and a specificity of 86.5%.

To assess whether underlying comorbidities influenced protein expression, we performed a multiple linear regression analysis. FINC, PF4, and CXCL7 levels were significantly decreased in patients with hematologic malignancies, while TIMP1 levels were elevated in those with chronic liver disease (Table S2). To further validate the robustness of our findings, we conducted a sensitivity analysis excluding patients with chronic liver disease and hematologic malignancies (*n* = 133). In this restricted cohort, ACTB and FINC remained the most discriminatory proteins between sepsis subgroups, each with a VIP score of approximately 3.5. PF4, CXCL7, and TIMP1 also retained discriminatory relevance (VIP > 1.5) (Figure S5). The six-variable model still showed high predictive performance for in-hospital mortality, with an AUROC of 0.869.

**Fig. 5:**
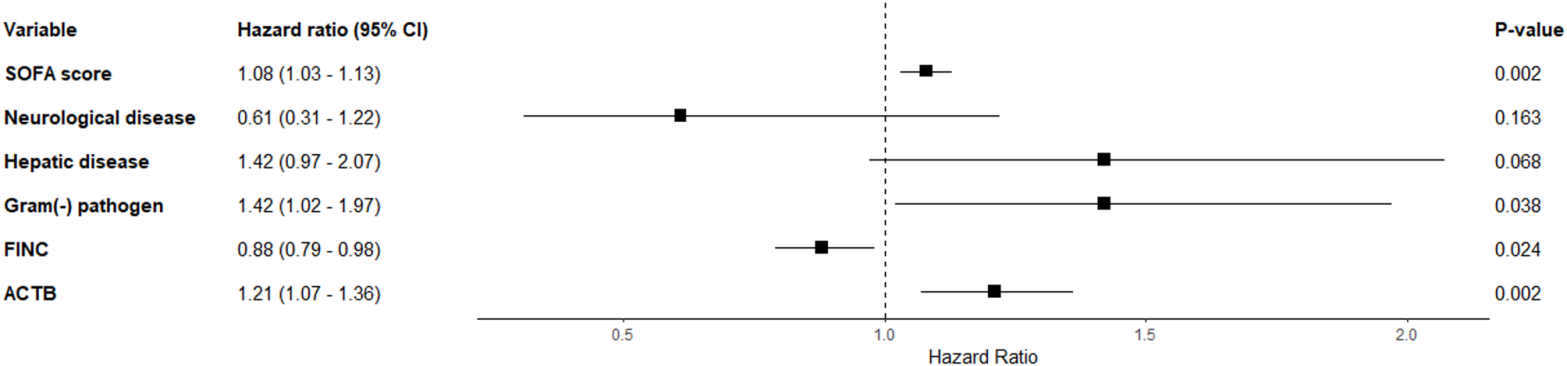
Forest plot of multivariable Cox-proportional hazards analysis for in-hospital mortality. Abbreviations: Gram (-), gram-negative; SOFA, Sequential Organ Failure Assessment; ACTB, β-actin; FINC, fibronectin

When the concentrations of CRP and the five top proteins were compared with the SOFA score and lactate levels, significant correlations were observed for all five proteins with the SOFA and lactate levels (all *p* < 0.001), except for CRP (Figure S6 & S7). Based on the univariable Cox proportional hazards analysis (Table S3), 15 variables with a *p*-value < 0.1 were identified and subsequently included in the multivariable Cox proportional hazards analysis. The multivariable analysis revealed that a higher SOFA score, increased ACTB levels, and gram-negative sepsis were associated with an increased risk of in-hospital mortality, with hazard ratios of 1.08 (*p* = 0.002), 1.21 (*p* = 0.002), and 1.42 (*p* = 0.038), respectively (Fig. 5). In addition, higher FINC levels were associated with a decreased risk of in-hospital mortality, with a hazard ratio [HR] of 0.88 (*p* = 0.024).

## Discussion

In this study, distinct proteomic profiles were identified when comparing sepsis patients to healthy controls and among sepsis subgroups according to patient outcomes. Although CRP demonstrated high diagnostic accuracy for sepsis, its prognostic utility within sepsis subgroups is limited. In contrast, ACTB, FINC, TIMP1, PF4, and CXCL7 showed greater potential for predicting sepsis outcomes. A six-variable model combining ACTB, FINC, TIMP1, PF4, CXCL7, and the SOFA score achieved the highest AUC of 0.903 for predicting in-hospital mortality. Increased ACTB and decreased FINC levels were significantly linked to in-hospital mortality, even after adjusting for clinical variables. This study emphasizes the significance of proteins associated with tissue injury, including cytoskeletal actin, in predicting sepsis outcomes.

The ACTB is a cytoplasmic actin which is essential for maintaining cell shape, motility, contractility, and regulating cellular signaling pathways [17, 18]. Actin plays a critical role in tissue regeneration and repair following wound injury [19, 20] and is also recognized as a damage-associated molecular pattern protein that triggers inflammatory cascades [21, 22]. In sepsis, this could lead to a cytokine storm and hyper-inflammatory state, exacerbating tissue damage and potentially causing multi-organ failure and death [23, 24]. Widespread cellular and tissue damage in patients with sepsis results in the release of intracellular actin into the bloodstream [25]. The release of actin from necrotic cells during sepsis poses significant risks, as actin filaments tend to polymerize, resulting in severe pathological effects within blood vessels. Circulating actin increases blood viscosity, disrupts microvascular flow, promotes platelet aggregation, induces microvascular thrombosis, and triggers pro-inflammatory mediator production [26, 27]. These toxic effects contribute to secondary tissue injury, worsening sepsis-associated organ dysfunction. Despite the biological significance of actin, few studies have measured actin levels in patients with sepsis, and none have investigated its prognostic value in this context. Lee et al. report that gelsolin deficiency and elevated plasma actin were linked to early sepsis [28]. Belsky et al. report significantly higher levels of actin levels in patients with septic shock than in healthy controls [18]. Our findings show that actin levels were elevated in patients with sepsis and increased with sepsis severity, particularly in patients who died early. Furthermore, significant correlations were found between actin levels and both the SOFA score, which reflects the degree of organ dysfunction, and lactate, a surrogate marker of tissue hypoxemia and shock severity produced by anaerobic cell metabolism [29]. These findings suggest that increased serum actin levels correlate with cellular and tissue damage, the severity of sepsis-related organ dysfunction, and a higher risk of mortality. This underscores the need for future research to develop therapeutic agents targeting actin to mitigate its harmful effects in sepsis.

The large glycoprotein FINC has been studied in the context of sepsis [30–32]. Soluble plasma FINC plays a critical role in wound healing, immune clearance of damaged tissue, removal of antibody-coated microorganisms, and phagocytosis [32]. Previous studies have reported its depletion in nonsurvivors with sepsis, which aligns with our findings [31, 33]. This reduction in circulating fibronectin levels during sepsis has been proposed to occur through multiple mechanisms, including decreased hepatic synthesis, intravascular consumption through proteolytic degradation, phagocytosis of opsonized particles, incorporation into fibrin clots during disseminated intravascular coagulation (DIC), and redistribution into the extravascular space where it binds to inflamed or injured tissue [31, 33]. Although a study reports the effect of FINC administration to improve sepsis outcomes, no significant effect was observed [34]. This study highlights that ACTB and FINC exhibit strong diagnostic accuracy in distinguishing patients with sepsis from healthy controls and predicting early mortality versus recovery in sepsis cases. Additionally, their AUC for predicting in-hospital mortality surpassed that of CRP and serum procalcitonin. Elevated serum actin and decreased FINC levels were significantly linked to increased in-hospital mortality in multivariate Cox proportional hazards analysis. These findings indicate that actin and FINC may serve as valuable diagnostic and prognostic biomarkers in sepsis.

The levels of TIMP1, PF4, and CXCL7 proteins varied across sepsis subgroups in this study. Matrix metalloproteinases (MMPs) are involved in extracellular matrix degradation and remodeling, and their inhibitor, such as TIMP1, regulates MMPs activity. Thus, maintaining a balance between MMPs and TIMP1 is essential for tissue integrity [35]. MMPs are upregulated in response to bacterial lipopolysaccharides or pro-inflammatory cytokines and regulate immune responses by facilitating leukocyte extravasation and recruitment to infection sites [36]. Studies show that the serum levels of TIMP1 and MMPs are associated with outcomes in patients with sepsis [37, 38]. Consistent with previous findings, this study demonstrated that TIMP1 levels were significantly higher in the early-death group, indicating a potential association with poor prognosis. However, no MMPs significantly differentiated sepsis subgroups based on outcomes.

PF4 and CXCL7 levels were decreased with worsening sepsis prognosis. These proteins, stored in platelet al.pha granules and released upon platelet activation at sites of vessel wall injury, are critical to immune response and coagulation during sepsis [39]. Platelets express Toll-like receptors that detect pathogen-associated molecular patterns, and this recognition triggers platelet activation and the release of alpha granules [39, 40]. Among the chemokines stored in platelet al.pha granules, PF4 and CXCL7 are the most abundant. This likely explains why only PF4 and CXCL7 showed significant differences between the healthy cohort and sepsis subgroups among platelet-derived granules. PF4, also known as chemokine ligand 4, is essential for neutrophil adhesion, degranulation, monocyte activation, and monocyte differentiation into macrophages and foam cells. CXCL7, cleaved into four distinct chemokines, is particularly significant for its active form, NAP-2, which promotes neutrophil chemotaxis and adhesion to endothelial cells [41, 42]. A few studies examined the PF4 and CXCL7 levels in patients with sepsis. The protective role of PF4 in infectious diseases is well-documented. Guo et al. highlight its importance in defending against influenza A virus in the lungs of mice [43], while Wegrzyn et al. show that patients with sepsis exhibit elevated levels of PF4 compared to that of healthy controls [44]. Among the patients with sepsis in the study by Wegrzyn, non-survivors and those with overt disseminated intravascular coagulation (DIC) display the lowest PF4 levels, suggesting a protective function mediated through macrophage activation. The findings of this study align with those of Wegrzyn, showing the lowest PF4 levels in patients with sepsis who died early. However, unlike the previous study, this analysis revealed that all sepsis subgroups exhibit decreased PF4 levels compared to that of healthy cohorts. These discrepancies may result from variations in sepsis severity, DIC prevalence, and infection types. Specifically, this study cohort had more severe conditions, with 77% experiencing septic shock and a mean SOFA score of 13.4, compared to 44.7% with septic shock and a mean SOFA score of 5.9 in the study by Wegrzyn. The mean platelet count in this study cohort was 97.2 × 10^3^/uL, indicating that the DIC with thrombocytopenia may contribute to the decreased PF4 levels observed in these patients. PF4 may be more extensively consumed in bacterial sepsis due to its binding to bacteria, aligning with the findings of Krystin et al., who show that PF4 binds to bacteria in a charge-dependent manner and enhances bacterial clearance through phagocytosis [45]. Regarding CXCL7, although one study reports its upregulation in septic rats [46], this study showed that CXCL7 levels were highest in healthy controls and declined with worsening sepsis outcomes. Although CXCL7 and PF4 may initially rise in response to bacterial invasion, their levels significantly decrease as DIC and sepsis-related thrombocytopenia progress, indicating worsening immune and coagulation dysfunction. Further research is needed to elucidate the roles and regulation of PF4 and CXCL7 in sepsis, particularly in bacterial infections and DIC.

This study compared the protein profiles between healthy controls and patients with sepsis and among sepsis subgroups, revealing distinct expression patterns. Sepsis groups showed significantly elevated levels of acute-phase reactants, including CRP, SAA1, and SAA2, reflecting the immediate immune response to infection of the host [47, 51]. While acute-phase proteins are valuable diagnostic markers for sepsis, their prognostic utility is limited [48–51]. Consistent with existing literature, this study showed no significant differences in acute-phase reactants levels across the outcome-based subgroups in patients with sepsis, as determined through several analytical methods. In contrast, proteins related to the wound healing [19, 20, 32, 35], such as ACTB, FINC, and TIMP1, showed significant upregulation or downregulation across sepsis subgroups. These findings suggest that severity of tissue injury is more pronounced in these patients and closely related to sepsis outcomes. In patients with septic shock, impaired oxygen delivery, endothelial barrier breakdown, and mitochondrial dysfunction lead to tissue injury and organ dysfunction [52], potentially driving the wound healing process observed in this analysis.

This study has some limitations. First, while SWATH-MS proteomics was used for protein quantification, complementary enzyme-linked immunosorbent assay (ELISA) validations were not performed. Consequently, the analysis was limited to the assessment of relative protein abundance, preventing absolute protein quantification. This limits direct comparisons across studies and reduces clinical applicability. Further research is required to validate these findings using standardized methods such as ELISA to enable absolute quantification and clinical application. Second, blood samples were collected only once at the time of sepsis diagnosis following ICU admission. This single time-point sampling limits the ability to tract dynamic protein level changes throughout the illness. Longitudinal measurements could provide deeper insights into sepsis progression and how biomarker utility for predicting outcomes evolves. Future studies incorporating longitudinal sampling are needed to validate and expand these findings, clarifying protein kinetics in relation to sepsis outcomes. Third, this study was conducted at a single tertiary hospital without external validation, which limits the generalizability of the findings. Although we strictly followed standard operating procedures for sample preparation and handling, such as the CLSI H3-A6 phlebotomy guideline [53] and the CLSI C64 guideline for quantitative protein analysis [54], residual hemolysis and protein degradation could not be fully quantified or excluded. Given the known sensitivity of plasma proteomics to pre-analytical variability, and the possibility of laboratory-specific bias or confounding, external validation in independent multicenter cohorts using standardized sample processing and orthogonal assays is warranted to confirm reproducibility and clinical applicability.

This study showed differences in serum proteomic profiles between patients with sepsis and healthy controls, as well as among sepsis subgroups. Although CRP and other acute-phase proteins effectively distinguished patients with sepsis from healthy individuals, they had limited ability to differentiate sepsis subgroups by outcomes. In contrast, a novel set of biomarkers, including ACTB, FINC, CXCL7, TIMP1, and PF4, was identified for sepsis prognosis. The six-variable model, incorporating SOFA scores, demonstrated high prognostic value for in-hospital mortality, potentially enabling better risk stratification for ICU patients with sepsis. Further studies with longitudinal samples and validation through complementary assays are needed to enhance the clinical applicability of these proteomic biomarkers.

## Supplementary Information

Below is the link to the electronic supplementary material.

Supplementary Material 1

## Data Availability

The datasets used and/or analyzed during the current study are available from the corresponding author on reasonable request.
