# Supplementary material for: Serum proteomes and their prognostic values in sepsis patients admitted to a medical intensive care unit: a single-center study using SWATH-MS proteomics
Source: Ann Intensive Care. 2025 Aug 27;15:126. doi: 10.1186/s13613-025-01543-y (PMC12391578; doi:10.1186/s13613-025-01543-y)
Supplement: Supplementary file 1 — Supplementary Material 1 [file 13613_2025_1543_MOESM1_ESM.docx]

**Serum Proteomes and their Prognostic Values in Sepsis Patients Admitted to a Medical Intensive Care Unit: A Single-Center Study using SWATH-MS proteomics**

**Supplementary materials**

**Appendix 1.** Proteomic sample collection, preparation and SWATH-MS analysis

**Appendix 2.** Proteomic data processing and statistical analyses

**Figure S1.** Proteomic profiles of healthy controls and sepsis patients according to outcome-based subgroups.

**Figure S2**. Concentrations of CRP and top five protein levels that can discriminate sepsis subgroups

**Figure S3**. ROC curves of six proteins discriminating 1) sepsis vs. healthy controls (green), 2) early death in patients with sepsis (red), 3) recovery in sepsis patients (blue), and 4) early death vs. recovery in patients with sepsis (purple)

**Figure S4.** ROC curves for the five-protein model and the SOFA and lactate model in predicting in-hospital mortality.

**Figure S5.** Proteomic profiles and ROC curve of the six-variable model in sepsis patients excluding those with chronic liver disease or hematologic malignancies (n = 133)

**Figure S6.** Correlation curves of six proteins with SOFA score

**Figure S7**. Correlation curves of six proteins with lactate

**Table S1.** Significantly different proteins between sepsis groups by Anova and Tukey’s honestly significant post hoc analysis

**Table S2.** Multiple Linear Regression analysis results of proteomics and underlying diseases

**Table S3.** Univariable Cox proportional hazard analysis of in-hospital mortality

**Appendix 1.** Proteomic sample collection, preparation and SWATH-MS analysis

1. Sample collection

All blood samples were collected and processed by highly experienced personnel, including research nurses and staff with over 10 years of experience, in accordance with established standard operating procedures. These procedures followed the Clinical and Laboratory Standards Institute (CLSI) H3-A6 guideline for phlebotomy [1] and the CLSI C64 guideline for the quantitative measurement of proteins and peptides by mass spectrometry [2]. Blood samples were centrifuged within 2 hours of collection, aliquoted to prevent repeated freeze–thaw cycles and stored at −80°C until analysis to preserve protein integrity and minimize degradation.

2. Protein digestion and sample preparation

Two microliter of plasma from patients was diluted 4-fold using phosphate buffered saline (PBS) and protein digestion was conducted using a suspension-trapping sample preparation approach [3]. The diluted plasma was resuspended in 400 μL of 5% SDS in 50 mM TEAB (pH 7.55), and disulfides were reduced using 20 mM dithiothreitol for 10 min at 95°C. Cysteines on protein were alkylated using 40 mM iodoacetamide in the dark for 30 min. The samples were diluted 10-fold in 12% phosphoric acid and each sample was then loaded onto a S-Trap mini column (ProtiFi, Farmingdale, NY, USA) along with 5 μg of Lys-C/trypsin mixture (Promega, Cat. No: V5071), and the column was incubated for 1 h at 47°C. The eluted peptide mixture was lyophilized with a cold trap and stored at -80°C until LC-MS analysis.

3. Liquid Chromatography and Mass spectrometry analysis for SWATH

Peptide samples were reconstituted in 0.1% formic acid and with the iRT-standard provided by the iRT-Kit (Biognosys AG, Schlieren, Switzerland) to be 1/10 dilution, according to the manufacturer’s instructions [4]. Each 20 μg sample was dissolved in a total volume of 20 μL. The injected samples (4 μL) were analyzed using a SCIEX TripleTOF 5600+ mass spectrometer system. For LC separation, the ekspertTM nanoLC 425 system (Eksigent, Dublin, CA, USA) was used with an Eksigent micro trap cartridge (ChromXP C18CL, 5 μm, 120 Å) as the trap column, and an Eksigent column (C18-CL, 0.3 × 150 mm, particle size 3 μm, pore size 120 Å) as the analytical column. The column temperature was maintained at 40°C. The samples were loaded onto the trap column using 100% eluent A (0.1% formic acid) at a flow rate of 10 μL/min. After 10 min, the peptide mixtures were separated for 57 min using eluent A and eluent B (0.1% formic acid in 100% acetonitrile). Eluent B was supplied at a flow rate of 5 μl/min and was increased from 3% to 25% over 38 min, 25% to 32% over 5 min, and 32% to 80% over 2 min, kept at 80% for 3 min, and then reduced to 3% over 1 min and kept at 3% for a further 8 min. For each sample, all mass spectrometry runs were operated in SWATH mode using 100 variable windows, as per the SCIEX technical notes. The SWATH parameters were as follows: lower m/z limit, 400; upper m/z limit, 1250; window overlap (Da), 1.0; CES, 5 for the smaller windows, 8 for the larger windows, and 10 for the largest windows. MS2 spectra were collected in the 100–1500 m/z range for 2.5 ms in the high sensitivity mode and the total cycle time was 2.8 s. Other MS parameters were set as follows: ion source gas 1 (GS1), 15; ion source gas 2 (GS2), 20; curtain gas (CUR), 30; temperature (TEM), 250°C; ion spray voltage floating (ISVF), 5500. Triplicate LC-MS/MS runs were analyzed, giving a total of 72 files (12 patients, two plasma samples, three replicates of each).

**Appendix 2.** Proteomic data processing and statistical analyses

The DIA files for individual samples from SWATH analysis were processed using Spectronaut (Version 13, Biognosis, USA) and compared with a pan-human protein mass spectrometry library [5]. Default analysis settings were used, with the following minor changes: the false discovery rate (FDR) of protein identification was set to 5% and a maximum of six precursors was used for quantification. For peptide/protein quantification, the peak area of fragment ions was selected, and the mean peptide quantity was measured. The resulting proteomic dataset was log_2_-transformed, normalized, and filtered to include values valid in at least 70% of one group. Missing values were imputed with random numbers drawn from a normal distribution with a width of 0.3 and down shift of 1.8. Statistical comparisons between groups were performed using Student’s t-tests with Benjamini–Hochberg correction, with a q-value < 0.1 considered significant.

Partial Least Squares Discriminant Analysis (PLS-DA) and Variable Importance Projection (VIP) scores, which measure the importance of variables in the PLS-DA model, were used to analyze differentially expressed proteins between groups. Hierarchical clustering heatmaps were generated using the Euclidean distance measure and Ward’s method. A fold change threshold of 2.0 and 0.5, along with a FDR of 0.05, were applied to generate the volcano plots. Clinical variables were presented as means with standard deviations or medians with interquartile ranges. Baseline patient characteristics and ICU treatments were compared across the three sepsis subgroups. The one-way analysis of variance (ANOVA) test or Kruskal–Wallis test was used for continuous variables, while the chi-squared or Fisher's exact test was used for categorical variables. For proteins with significantly different log_2_abundances in the ANOVA test, Tukey’s honestly significant difference post hoc analysis was performed. Receiver operating characteristic (ROC) curve analysis was performed to evaluate the discriminating power of the proteins. To assess the influence of comorbidities on protein levels, multiple linear regression analysis was also conducted. Pearson's correlation analysis was used to compare the log_2_abundance of proteins with the SOFA score, and the correlation coefficient (R) was calculated accordingly. Risk factors for in-hospital mortality were analyzed using Cox proportional hazards analysis. Variables with p-values < 0.1 in the univariable analysis were included in the multivariable analysis through backward elimination. All p-values were two-tailed, with statistical significance defined as a p-value of < 0.05. Proteomic data processing was performed using Perseus software v2.1.2.0 (<https://maxquant.net/perseus>) [6], and both proteomic and clinical variables were analyzed using MetaboAnalyst 6.0 (<https://www.metaboanalyst.ca>) [7] and R 4.2.1 (R Core Team, Vienna, Austria).

**Figure S1.** Proteomic profiles of healthy controls and sepsis patients according to outcome-based subgroups. **
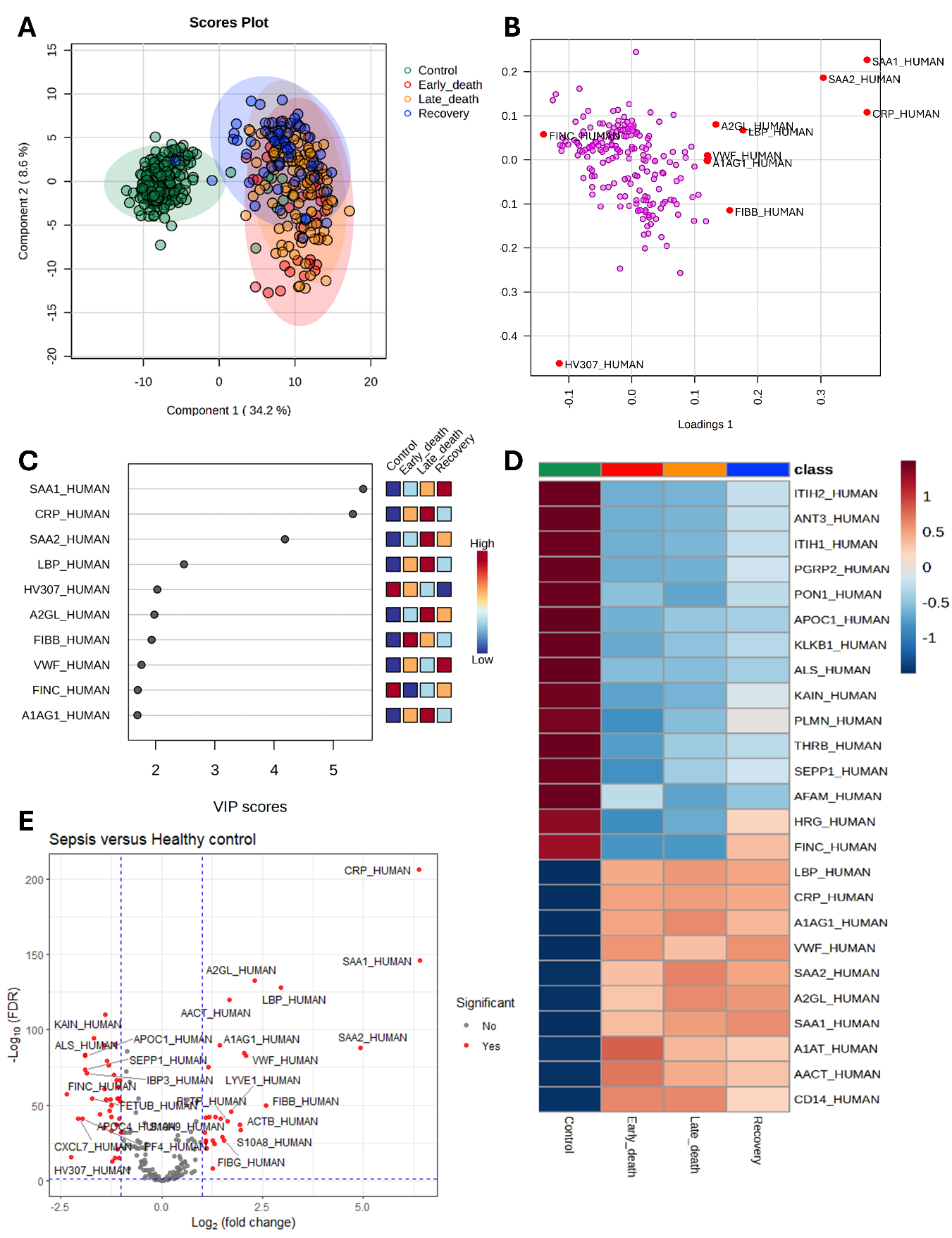
**

(A) Partial least squares discriminant analysis (PLS-DA) based on protein abundance in all samples. Overall, 509 samples from patients with sepsis and healthy controls were analyzed, with 189 proteins included in the dataset after data processing. PLS-DA revealed a gradient along Component 1, distinguishing healthy controls from the sepsis groups. Additionally, the sepsis subgroups were clearly separated along Component 2. (B) Protein loadings. Acute phase proteins, including C-reactive protein (CRP), serum amyloid A1 (SAA1), and serum amyloid A2 (SAA2), exhibited high positive loadings on both components 1 and 2. (C) Variable importance in projection (VIP) score for top 10 proteins. VIP scores analysis revealed that SAA1, CRP, and SAA2 had the highest VIP scores. (D) Hierarchical heatmap for top 25 discriminating proteins. A heatmap of mean protein abundance in sepsis groups and healthy controls revealed no significant differences in CRP, SAA1, and SAA2 levels across sepsis subgroups, and their mean values did not correlate with sepsis outcomes. (E) Volcano plot for differential protein abundance between sepsis versus healthy control. The volcano plot demonstrated that CRP, SAA1, and SAA2 were expressed at higher levels in patients with sepsis than in healthy controls

**Figure S2**. Concentrations of CRP and top five protein levels that can discriminate sepsis subgroups


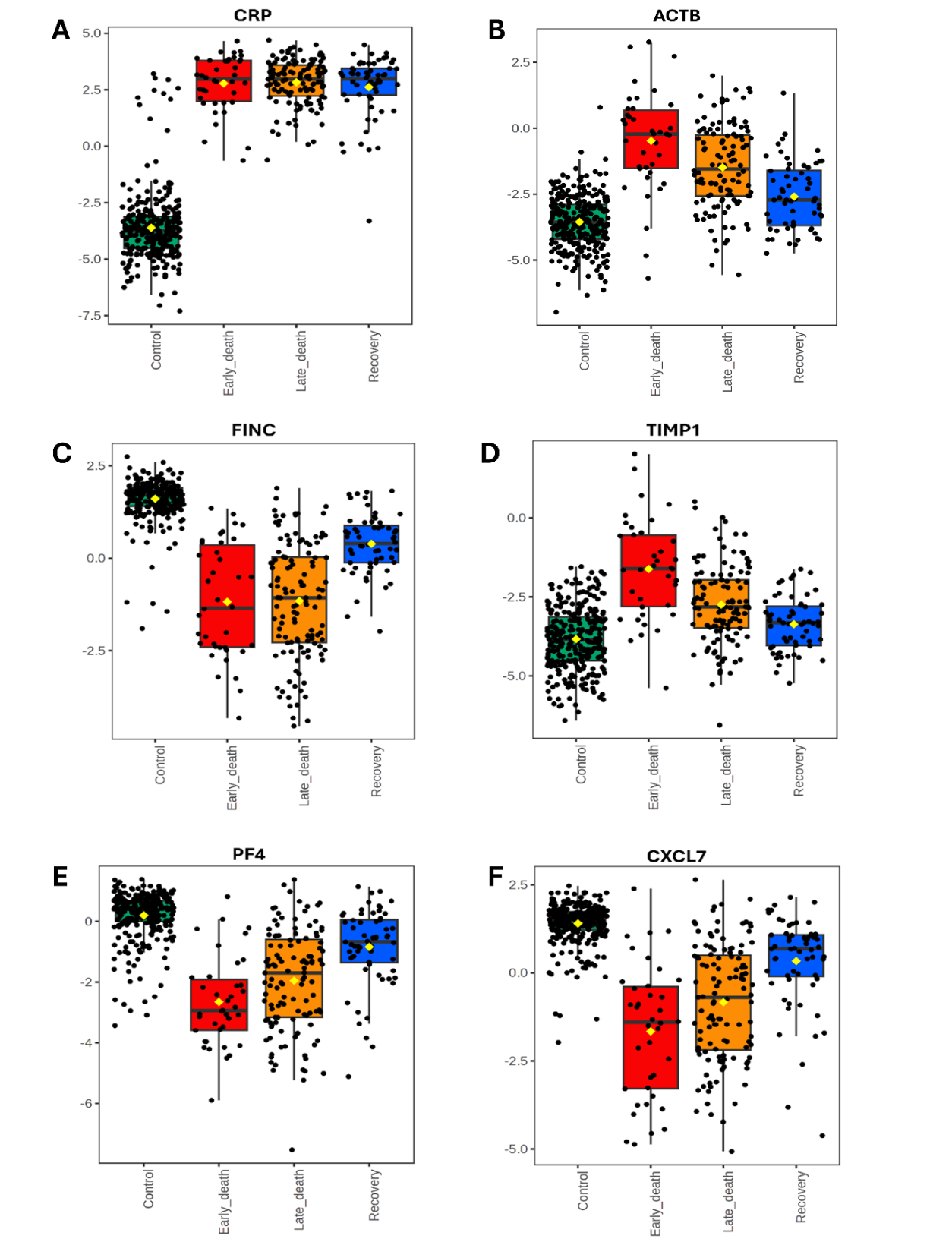


**Figure S3**. ROC curves of six proteins discriminating 1) sepsis vs. healthy controls (green), 2) early death in patients with sepsis (red), 3) recovery in sepsis patients (blue), and 4) early death vs. recovery in patients with sepsis (purple)


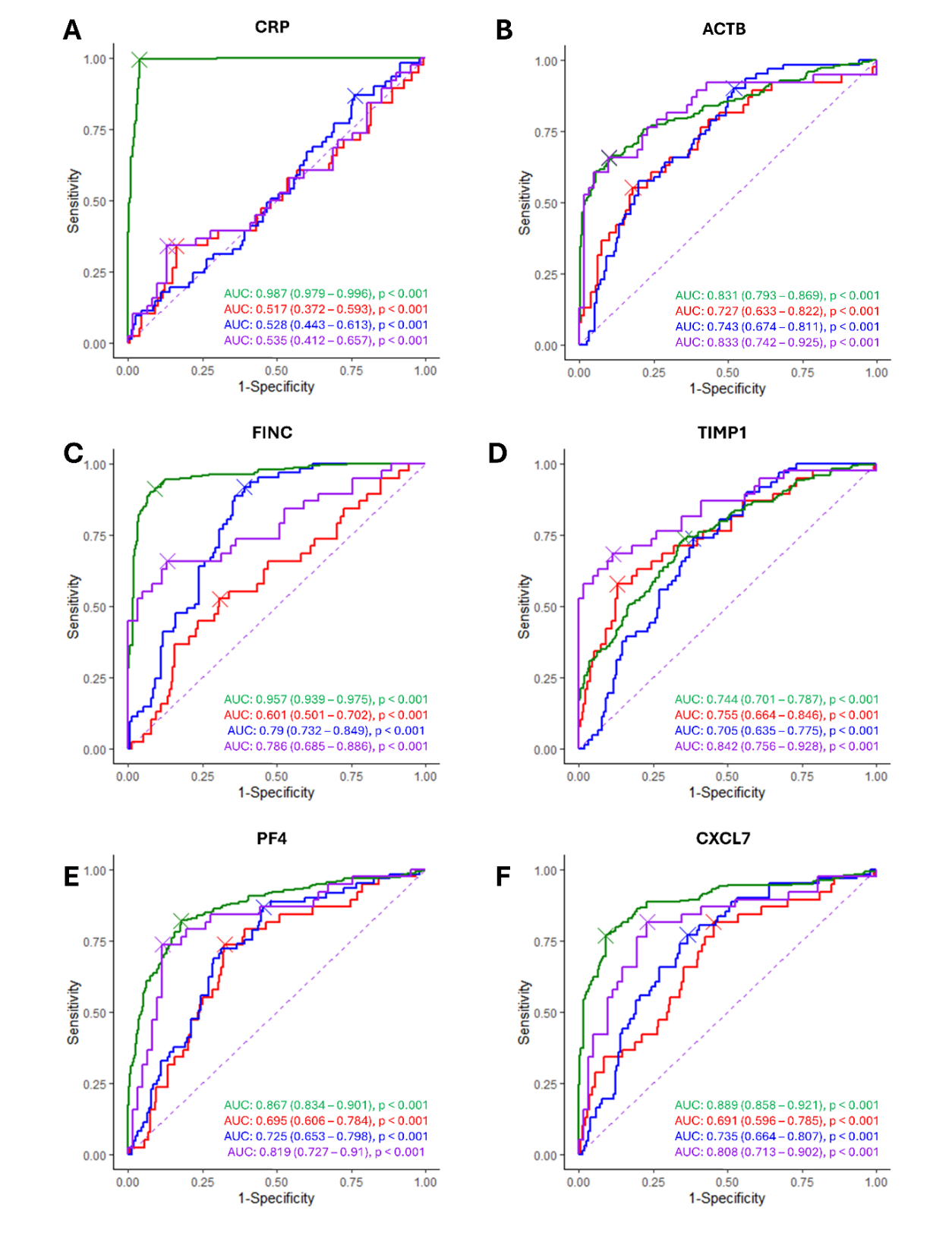


While CRP demonstrated an excellent discriminate power with an AUC of 0.987 (95% CI: 0.979–0.996, p < 0.001), CRP showed poor performance in distinguishing between sepsis subgroups, with AUC values ranging from 0.517–0.535

Abbreviations: AUC, area under the curve; ROC, receiver operative characteristic; CRP, c-reactive protein; ACTB, β-actin; FINC, fibronectin; CXCL7, C-X-C motif chemokine 7; PF4, platelet factor 4; TIMP1, Metalloproteinase Inhibitor 1

**Figure S4.** ROC curves for the five-protein model and the SOFA and lactate model in predicting in-hospital mortality.

**
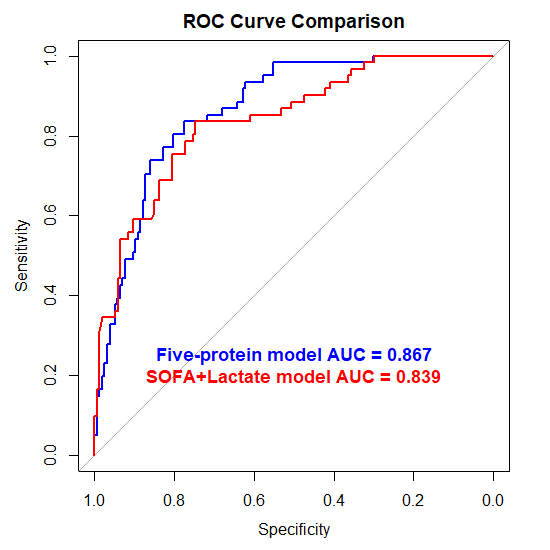
**

Abbreviations: ROC, receiver operating characteristic; SOFA, Sequential Organ Failure Assessment; AUC, area under the curve

**Figure S5.** Proteomic profiles and ROC curve of the six-variable model in sepsis patients excluding those with chronic liver disease or hematologic malignancies (n = 133)

**
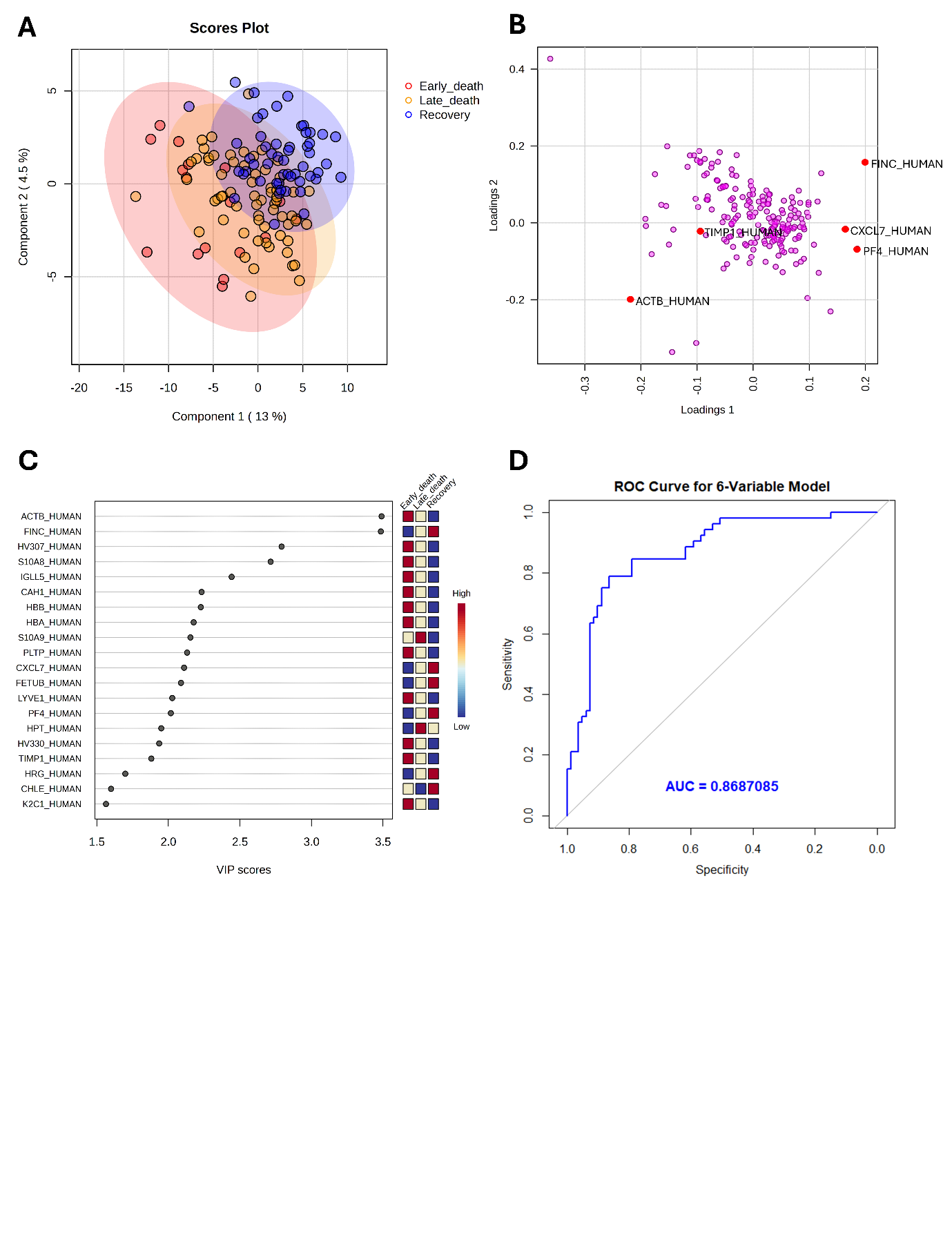
**

(A) PLS-DA score plot based on protein abundance, (B) PLS-DA loading plot, (C) VIP scores for the top 15 discriminatory proteins, (D) ROC curve of the six-variable model for predicting in-hospital mortality

Abbreviations: PLS-DA, partial least squares discriminant analysis; VIP, variable importance in projection; ROC, receiver operating characteristic; AUC, area under the curve

**Figure S6.** Correlation curves of six proteins with SOFA score


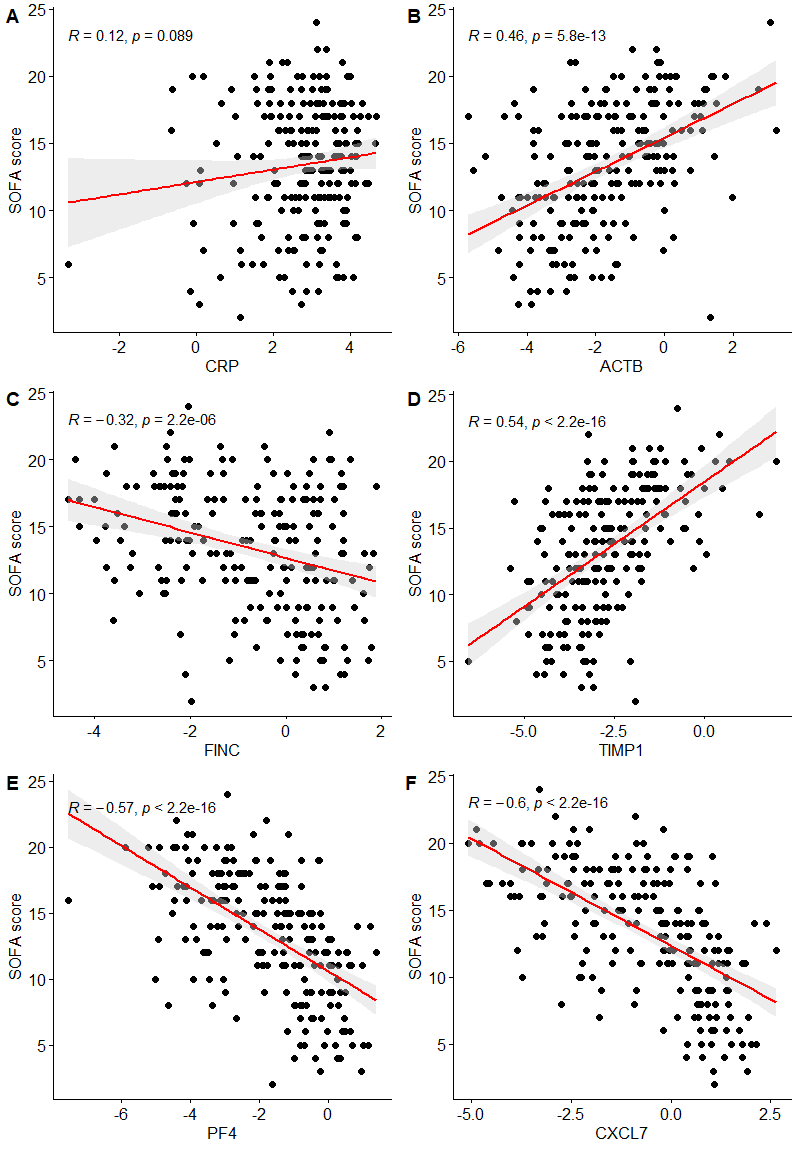


Abbreviations: SOFA, Sequential Organ Failure Assessment; CRP, c-reactive protein; ACTB, β-actin; FINC, fibronectin; CXCL7, C-X-C motif chemokine 7; PF4, platelet factor 4; TIMP1, Metalloproteinase Inhibitor 1

**Figure S7**. Correlation curves of six proteins with lactate


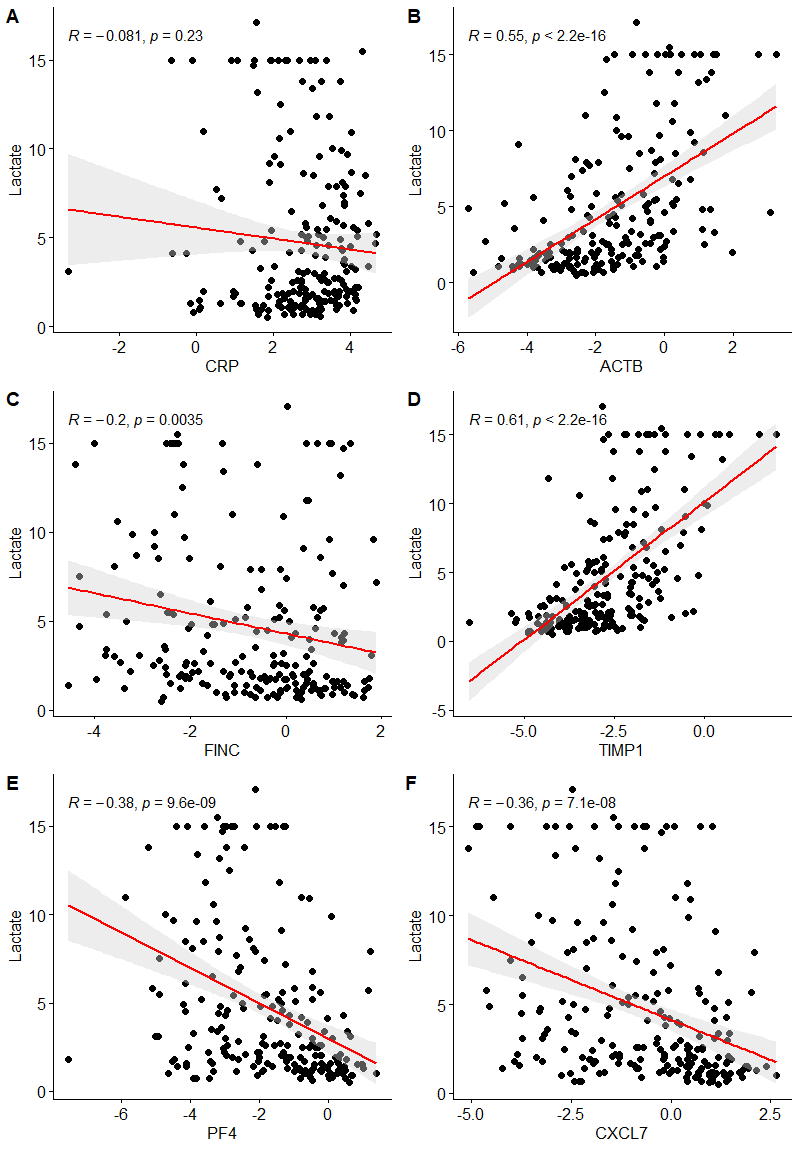


Lactate is measured in units of mg/dL; Abbreviations: CRP, c-reactive protein; ACTB, β-actin; FINC, fibronectin; CXCL7, C-X-C motif chemokine 7; PF4, platelet factor 4; TIMP1, Metalloproteinase Inhibitor 1

**Table S1.** Significantly different proteins between sepsis groups by Anova and Tukey’s honestly significant post hoc analysis

| Proteins | -Log ANOVA  p-value | ANOVA  q-value | Tukey's HSD |
| --- | --- | --- | --- |
| FINC_HUMAN | 10.5368 | 0 | Recovery_Early_death;Recovery_Late_death |
| TIMP1_HUMAN | 9.45728 | 0 | Late_death_Recovery;Early_death_Recovery;Early_death_Late_death |
| ACTB_HUMAN | 8.84917 | 0 | Late_death_Recovery;Early_death_Recovery;Early_death_Late_death |
| HRG_HUMAN | 8.34495 | 0 | Recovery_Early_death;Recovery_Late_death |
| CXCL7_HUMAN | 7.66037 | 0 | Late_death_Early_death;Recovery_Early_death;Recovery_Late_death |
| PF4_HUMAN | 7.06618 | 0 | Late_death_Early_death;Recovery_Early_death;Recovery_Late_death |
| VTNC_HUMAN | 6.92637 | 0 | Late_death_Early_death;Recovery_Early_death;Recovery_Late_death |
| PLTP_HUMAN | 6.74044 | 0.000444444 | Late_death_Recovery;Early_death_Recovery;Early_death_Late_death |
| PLMN_HUMAN | 5.73491 | 0.004 | Recovery_Early_death;Recovery_Late_death |
| CFAB_HUMAN | 5.40755 | 0.00581818 | Late_death_Early_death;Recovery_Early_death;Recovery_Late_death |
| LYVE1_HUMAN | 5.35277 | 0.00566667 | Late_death_Recovery;Early_death_Recovery |
| CYTC_HUMAN | 4.72141 | 0.0110769 | Early_death_Recovery;Early_death_Late_death |
| HBB_HUMAN | 4.63903 | 0.0117143 | Late_death_Recovery;Early_death_Recovery |
| IC1_HUMAN | 4.47462 | 0.0125333 | Late_death_Recovery;Early_death_Recovery |
| THBG_HUMAN | 4.40343 | 0.01275 | Recovery_Early_death;Recovery_Late_death |
| HBA_HUMAN | 4.33424 | 0.0127059 | Late_death_Recovery;Early_death_Recovery |
| HBB_HUMAN | 4.2289 | 0.0135556 | Late_death_Recovery;Early_death_Recovery |
| CO8A_HUMAN | 3.97316 | 0.0178947 | Late_death_Early_death;Recovery_Early_death;Recovery_Late_death |
| FIBB_HUMAN | 3.83509 | 0.0218 | Late_death_Recovery;Early_death_Recovery;Early_death_Late_death |
| FIBG_HUMAN | 3.71208 | 0.0232727 | Late_death_Recovery;Early_death_Recovery;Early_death_Late_death |
| VTDB_HUMAN | 3.66689 | 0.024 | Recovery_Early_death;Recovery_Late_death |
| KAIN_HUMAN | 3.66043 | 0.023 | Recovery_Early_death;Recovery_Late_death |
| CO8B_HUMAN | 3.64149 | 0.02256 | Late_death_Early_death;Recovery_Early_death |
| FETUB_HUMAN | 3.51418 | 0.0249231 | Recovery_Early_death;Recovery_Late_death |
| HABP2_HUMAN | 3.3861 | 0.0294815 | Late_death_Early_death;Recovery_Early_death |
| CO9_HUMAN | 3.23599 | 0.0384286 | Late_death_Early_death;Recovery_Early_death |
| HEP2_HUMAN | 3.15385 | 0.0423448 | Recovery_Early_death;Recovery_Late_death |

Abbreviations: ANOVA, analysis of variance

**Table S2.** Multiple Linear Regression analysis results of proteomics and underlying diseases

|  | ACTB | | FINC | | PF4 | | CXCL7 | | TIMP1 | |
| --- | --- | --- | --- | --- | --- | --- | --- | --- | --- | --- |
|  | beta | p | beta | p | beta | p | beta | p | beta | p |
| Cardiovascular disease | 0.08761 | 0.768 | 0.006043 | 0.983 | 0.01767 | 0.949 | 0.06734 | 0.812 | 0.22579 | 0.275 |
| Chronic lung disease | 0.27820 | 0.360 | 0.068450 | 0.821 | 0.33661 | 0.235 | 0.19120 | 0.508 | -0.22586 | 0.284 |
| Chronic neurologic disease | -0.30241 | 0.382 | 0.457642 | 0.186 | 0.31730 | 0.326 | 0.12330 | 0.708 | -0.67605 | 0.005 |
| Chronic liver disease | 0.03861 | 0.878 | 0.331095 | 0.187 | -0.12386 | 0.597 | -0.08546 | 0.721 | 0.47979 | 0.006 |
| Diabetes | -0.20887 | 0.350 | -0.266986 | 0.232 | -0.30748 | 0.141 | -0.08520 | 0.688 | -0.27699 | 0.075 |
| Chronic kidney disease | 0.09886 | 0.772 | -0.050927 | 0.881 | -0.13432 | 0.674 | -0.35682 | 0.274 | -0.01448 | 0.951 |
| Hematological  malignancies | 0.02298 | 0.938 | -0.983567 | 0.001 | -1.07781 | 0.0001 | -1.27227 | <0.0001 | 0.15151 | 0.464 |
| Solid malignant tumors | 0.17227 | 0.425 | -0.229832 | 0.291 | 0.11328 | 0.574 | 0.05061 | 0.806 | 0.03301 | 0.826 |
| SOFA | 0.08246 | 0.004 | -0.096643 | 0.001 | -0.15126 | <0.0001 | -0.18544 | <0.0001 | 0.05666 | 0.004 |
| Lactate | 0.16463 | <0.0001 | -0.007961 | 0.776 | -0.03480 | 0.183 | -0.01941 | 0.466 | 0.13694 | <0.0001 |

Abbreviation: SOFA, Sequential Organ Failure Assessment; ACTB, β-actin; FINC, fibronectin; CXCL7, C-X-C motif chemokine 7; PF4, platelet factor 4; TIMP1, Metalloproteinase Inhibitor 1

**Table S3.** Univariable Cox proportional hazard analysis of in-hospital mortality

|  | Hazard Ratio (95% CI) | P-value |
| --- | --- | --- |
| Age | 0.99 (0.98–1.00) | 0.05 |
| Male sex | 0.77 (0.55–1.07) | 0.12 |
| BMI | 0.99 (0.94–1.03) | 0.5 |
| Underlying diseases |  |  |
| Cardiovascular disease | 0.83 (0.52–1.31) | 0.4 |
| Chronic lung disease | 0.99 (0.61–1.59) | > 0.9 |
| Chronic neurological disease | 0.465 (0.24–0.88) | 0.019 |
| Chronic liver disease | 1.99 (1.42–2.79) | < 0.001 |
| Diabetes | 0.94 (0.67–1.31) | 0.7 |
| Chronic kidney disease | 1.20 (0.72–1.98) | 0.5 |
| Hematologic malignancies | 1.81 (1.22–2.68) | 0.003 |
| Solid malignant tumors | 1.00 (0.72–1.39) | > 0.9 |
| Site of infection |  |  |
| Pulmonary | 1.14 (0.83–1.57) | 0.4 |
| Abdominal | 0.99 (0.67–1.45) | > 0.9 |
| Urinary | 0.68 (0.21–2.14) | 0.5 |
| Skin soft tissue | 0.69 (0.37–1.28) | 0.2 |
| Catheter | 0.63 (0.20–1.99) | 0.4 |
| Systemic | 1.48 (0.82–2.68) | 0.2 |
| Central nervous system | 0.86 (0.21–3.49) | 0.8 |
| Endocarditis | 1.30 (0.48–3.51) | 0.6 |
| Septic shock | 1.34 (0.90–2.01) | 0.15 |
| SOFA score | 1.15 (1.10–1.20) | < 0.001 |
| Lactate | 1.11 (1.08–1.15) | < 0.001 |
| Bacteremia | 0.89 (0.65–1.23) | 0.5 |
| Gram-negative pathogen | 1.46 (1.06–2.01) | 0.021 |
| ICU treatment |  |  |
| Mechanical ventilation | 2.63 (1.49–4.66) | < 0.001 |
| Renal replacement therapy | 1.67 (1.21–2.31) | 0.002 |
| Adjuvant steroids | 1.48 (1.04–2.10) | 0.030 |
| Proteins |  |  |
| ACTB | 1.41 (1.27–1.57) | < 0.001 |
| FINC | 0.79 (0.71–0.87) | < 0.001 |
| TIMP1 | 1.55 (1.36–1.78) | < 0.001 |
| PF4 | 0.80 (0.73–0.87) | < 0.001 |
| CXCL7 | 0.81 (0.75–0.89) | < 0.001 |

Abbreviations: BMI, body mass index; SOFA, sequential organ failure assessment; ICU, intensive care unit; CRP, c-reactive protein; ACTB, β-actin; FINC, fibronectin; CXCL7, C-X-C motif chemokine 7; PF4, platelet factor 4; TIMP1, Metalloproteinase Inhibitor 1

Reference

1. Clinical Laboratory Standards Institute. *Procedures for collection of diagnostic blood specimens by venipuncture; approved guideline*, 6th ed. CLSI document H3-A6. CLSI: Wayne, PA, 2007.

2. Clinical Laboratory Standards Institute. *Quantitative Measurement of Proteins and Peptides by Mass Spectrometry.* 1st ed. CLSI guideline C64. Clinical and Laboratory Standards Institute; 2021.

3. HaileMariam M, Eguez RV, Singh H, Bekele S, Ameni G, Pieper R, et al. S-Trap, an Ultrafast Sample-Preparation Approach for Shotgun Proteomics. J Proteome Res. 2018;17(9):2917-24. Epub 20180828. doi: 10.1021/acs.jproteome.8b00505. PubMed PMID: 30114372.

4. Escher C, Reiter L, MacLean B, Ossola R, Herzog F, Chilton J, et al. Using iRT, a normalized retention time for more targeted measurement of peptides. Proteomics. 2012;12(8):1111-21. doi: 10.1002/pmic.201100463. PubMed PMID: 22577012; PubMed Central PMCID: PMCPMC3918884.

5. Zhu T, Zhu Y, Xuan Y, Gao H, Cai X, Piersma SR, et al. DPHL: A DIA Pan-human Protein Mass Spectrometry Library for Robust Biomarker Discovery. Genomics Proteomics Bioinformatics. 2020;18(2):104-19. Epub 20200812. doi: 10.1016/j.gpb.2019.11.008. PubMed PMID: 32795611; PubMed Central PMCID: PMCPMC7646093.

6. Tyanova S, Temu T, Sinitcyn P, Carlson A, Hein MY, Geiger T, et al. The Perseus computational platform for comprehensive analysis of (prote)omics data. Nat Methods. 2016;13(9):731-40. Epub 20160627. doi: 10.1038/nmeth.3901. PubMed PMID: 27348712.

7. Pang Z, Lu Y, Zhou G, Hui F, Xu L, Viau C, et al. MetaboAnalyst 6.0: towards a unified platform for metabolomics data processing, analysis and interpretation. Nucleic Acids Res. 2024;52(W1):W398-w406. doi: 10.1093/nar/gkae253. PubMed PMID: 38587201; PubMed Central PMCID: PMCPMC11223798.
